# Replication origin location might contribute to genetic variability in *Trypanosoma cruzi*

**DOI:** 10.1186/s12864-020-06803-8

**Published:** 2020-06-22

**Authors:** Christiane Bezerra de Araujo, Julia Pinheiro Chagas da Cunha, Davi Toshio Inada, Jeziel Damasceno, Alex Ranieri Jerônimo Lima, Priscila Hiraiwa, Catarina Marques, Evonnildo Gonçalves, Milton Yutaka Nishiyama-Junior, Richard McCulloch, Maria Carolina Elias

**Affiliations:** 1 Laboratório de Ciclo Celular, Instituto Butantan, São Paulo, Brazil; 2 Center of Toxins, Immune Response and Cell Signaling (CeTICS), Instituto Butantan, São Paulo, Brazil; 3 The Wellcome Centre for Molecular Parasitology, Institute of Infection, Immunity and Inflammation, University of Glasgow, Glasgow, UK; 4 Laboratório de Tecnologia Biomolecular – Bioinformática, Instituto de Ciências Biológicas, Universidade Federal do Pará, Belém, Brazil; 5 Fundação Oswaldo Cruz, Instituto Carlos Chagas, Paraná, Brazil; 6 Laboratório Especial de Toxinologia Aplicada, Instituto Butantan, São Paulo, Brazil

**Keywords:** Replication origins, *Trypanosoma cruzi*, Genetic variability, DGF-1

## Abstract

**Background:**

DNA replication in trypanosomatids operates in a uniquely challenging environment, since most of their genomes are constitutively transcribed. *Trypanosoma cruzi*, the etiological agent of Chagas disease, presents high variability in both chromosomes size and copy number among strains, though the underlying mechanisms are unknown.

**Results:**

Here we have mapped sites of DNA replication initiation across the *T. cruzi* genome using Marker Frequency Analysis, which has previously only been deployed in two related trypanosomatids. The putative origins identified in *T. cruzi* show a notable enrichment of GC content, a preferential position at subtelomeric regions, coinciding with genes transcribed towards the telomeres, and a pronounced enrichment within coding DNA sequences, most notably in genes from the Dispersed Gene Family 1 (DGF-1).

**Conclusions:**

These findings suggest a scenario where collisions between DNA replication and transcription are frequent, leading to increased genetic variability, as seen by the increase SNP levels at chromosome subtelomeres and in DGF-1 genes containing putative origins.

## Background

Genome replication is responsible for accurate transmission of genetic information through cell division cycles. As originally proposed in 1963, cells rely on two genetic elements to duplicate their genome: the replicator, a DNA region where replication begins (now named the replication origin), and the initiator, a protein or a protein complex that recognizes the replicator [1]. Specific DNA sequences to define the replicator are found in the genomes of bacteria, some archaea [2], and in *Saccharomyces cerevisiae* and related species [3]. In all other eukaryotes, clear consensus sequences for origins are elusive, perhaps indicating there are no cis elements to initiate replication in most of these cells and organisms. Instead, association of the initiator with origins may be dictated by nuclear architecture, gene density, chromatin status (such as histone modification or nucleosome positioning), transcriptional activity, and AT or CG content [4–6].

In eukaryotes, the initiator is termed the Origin Recognition Complex (ORC) [7, 8] and is assembled at replication origins during mitosis-G1 phases of the cell cycle, when it recruits, via Cdc6 and Cdt1, the MCM replication helicase, allowing origins become ‘licensed’ [9, 10]. Then, when cells reach S-phase, a set of enzymatic and regulatory factors activate some, but not all, origins, which are differently used depending on the cell types and stages of development, and even in different cells of the same population. According to the usage, origins have been classified as constitutive (fired at same position in different cells of a population), flexible (fired stochastically in different cells) and dormant (fired as consequence of replication stress) [5]. After origin firing, bidirectional replication forks travel until they reach termination sites. However, the replisome can be stalled by depletion of nucleotide pools or barriers on the template, such as DNA damage, secondary structures or protein complexes [11]. Collision between replication and transcription is considered especially problematic, as each are catalyzed by large multiprotein machines, and can occur co-directionally, when the replication fork and transcription machinery are moving in the same direction, or on the leading strand and is head-on, when the fork and transcription are moving towards each other. Accumulated evidence suggests head-on collisions have a more pronounced effect on genome instability, perhaps because more extensive changes to the machinery and template are needed to resolve such conflicts [12], some of which might result in increased single-stranded DNA gaps and DNA double strand break (DSB) formation [13]. Alternatively, genome instability may arise because stalled replisomes promote the action of translesion DNA polymerases (Pols) [14], which can catalyze error-prone DNA synthesis [15]. Taken together, considerably greater flexibility in initiator-directed origin usage is found in eukaryotes than in prokaryotes. Whether such flexibility might extend yet further, and perhaps include transcription-driven processes [16] is less clear.

The Trypanosomatida is a grouping of single-celled eukaryotic that includes the human pathogens, *Leishmania spp.*, *Trypanosoma cruzi* and *Trypanosoma brucei*, which are responsible for more than 50,000 deaths annually [17]. Understanding DNA replication in these organisms is not only important to comprehend how parasite proliferation is controlled, but the highly unusual manner in which they express their genes suggests the potential for unparalleled interaction, and potentially conflicting, between replication and transcription. Virtually every trypanosomatid protein-coding gene is found within a directional gene cluster (DGC) that can contain hundreds of genes with the same orientation [18]. This organization reflects transcription, where all genes within a DGC are transcribed from a single RNA pol II transcription initiation site, producing multigene pre-mRNAs that are processed to generate mature mRNAs through trans-splicing and polyadenylation [19]. The pervasive, highly directional movement of RNA pol II across the genome appears common to all kinetoplastids [20], far surpassing multigenic transcription described in other eukaryotes, and has already prompted investigations concerning DNA replication initiation and coordination with transcription in *T. brucei* and *Leishmania major*. In *T. brucei* origins were mapped by marker frequency analysis sequence (MFA-seq; sort-seq in yeast) [21] coupled to ChIP analysis of one component of ORC [22], termed ORC1/CDC6 [23]. These data show that all mapped *T. brucei* origins are found at the boundaries of the DGCs, which all appear to bind ORC1/CDC6. Nonetheless, not all ORC1/CDC6 sites are activated as origins, resulting in very widely spaced origins. Furthermore, the frequency of origin use, or the timing of activation, is variable across the genome, but the pattern of initiation mapped by MFA-seq displays considerable inflexibility during life cycle progression or growth [24]. Nonetheless, there are connections between transcription and replication: MFA-seq data shows DNA replication is more strongly impeded as it meets transcription head-on [23], RNAi of ORC1/CDC6 increases transcript abundance at the start and end of the DGCs (Tiengwe et al., 2012), telomere transcription levels influence replication timing, and telomere variation is dependent on ORC1/CDC6 levels [25]. MFA-seq suggests a pronounced difference in origin usage in *Leishmania* compared with *T. brucei*, with only a single origin per chromosome detected [26]. Such a difference is perhaps surprising, given the common use of multigenic transcription, and the fact the mapped origins were found, like in *T. brucei*, at the ends of DGCs (with ~ 40% of locations conserved relative to *T. brucei*). However, recent data perhaps suggest greater flexibility in trypanosomatid replication than is suggested by MFA-seq. In *T. brucei*, analysis of one chromosome suggests the activation of at least one back-up origin after hydroxyurea-induced impairment of DNA replication [27]. Indeed, DNA combing in both *T. brucei* and *Leishmania* was used to extrapolate a greater number of predicted sites of DNA replication initiation than origins mapped by MFA-seq, though was not able determine if these predicted sites relate to ORC binding or, indeed, in what part of the genome they might reside [28]. Finally, mapping nascent DNA strands in *L. major* suggested > 5000 sites of DNA replication initiation, with very limited correlation with the ends of the DGCs [29]. Taken as a whole, these studies might indicate that complete replication of the entire genome in the relatively short S phases of *T. brucei* and *Leishmania* [30] may require not merely constitutive origins, but also further flexible and/or dormant origins, which might or might not coincide with ORC binding. Moreover, intersection between transcription and replication may not simply be at DGC boundaries [23], but sites of replication initiation may also be located where RNA Pol II stalls or slows down during traversal of a DGC [29]. Thus, multigenic transcription may be an important determinant of DNA replication, allowing co-ordinated recruitment of ORC and RNA Pol [23], or using transcription pausing [29] or replication-transcription clashes [31] to promote DNA replication initiation.

A limitation of the emerging data discussed above is that so far only two members of the trypanosomatid grouping have been examined, meaning the basis for the differences in DNA replication in *T. brucei* and *Leishmania* remain unclear. Here, we seek to address these limitations by mapping DNA replication in *T. cruzi*, widening the range of parasites, and genome organization, examined. The success of *T. cruzi* infection is in part due to 18% of its genome that is composed of multigene families, including both functional and pseudogenes predicted to encode surface proteins that contribute to cellular invasion and escape from immunity [32, 33]. Among these families, the trans-sialidase (TS) and dispersed gene family-1 (DGF-1) genes are enriched at subtelomeric regions of chromosomes [33], though other gene arrays are found throughout the chromosomes. Intriguingly, next generation sequencing has suggested that *T. cruzi*, like *Leishmania*, displays chromosomes that deviate from diploidy [34], whereas no such aneuploidy is seen in *T. brucei* [35]. Whether this difference relates to *T. cruzi* and *Leishmania* each having their genome housed in large numbers (> 35) of relatively small chromosomes, whereas the *T. brucei* genome is found in 11 relatively large chromosomes, is unknown. Nonetheless, in the last years, evidence has accumulated suggesting that gene arrays are sites that favor homologous recombination (HR) [36–38] as the driver of genetic variability among these families [38]. However, how HR is triggered in these locations, is still unknown.

Here we used MFA-seq to provide the first genome-wide map of DNA replication initiation sites in *T. cruzi*. Despite the technical challenge of next generation sequence mapping in this uniquely repetitive genome, we show that some sites of replication initiation map to the borders of the DGCs, as seen for MFA-seq mapping in *T. brucei* and *Leishmania* and therefore suggesting a widespread, conserved localization of origins. In addition, we provide evidence that DNA replication initiation is also frequently being located at DGF-1 genes, which may explain the high genetic variability observed in such gene families.

## Results

### *T. cruzi* DNA replication origins were determined by two different approaches

**Fig. 1:**
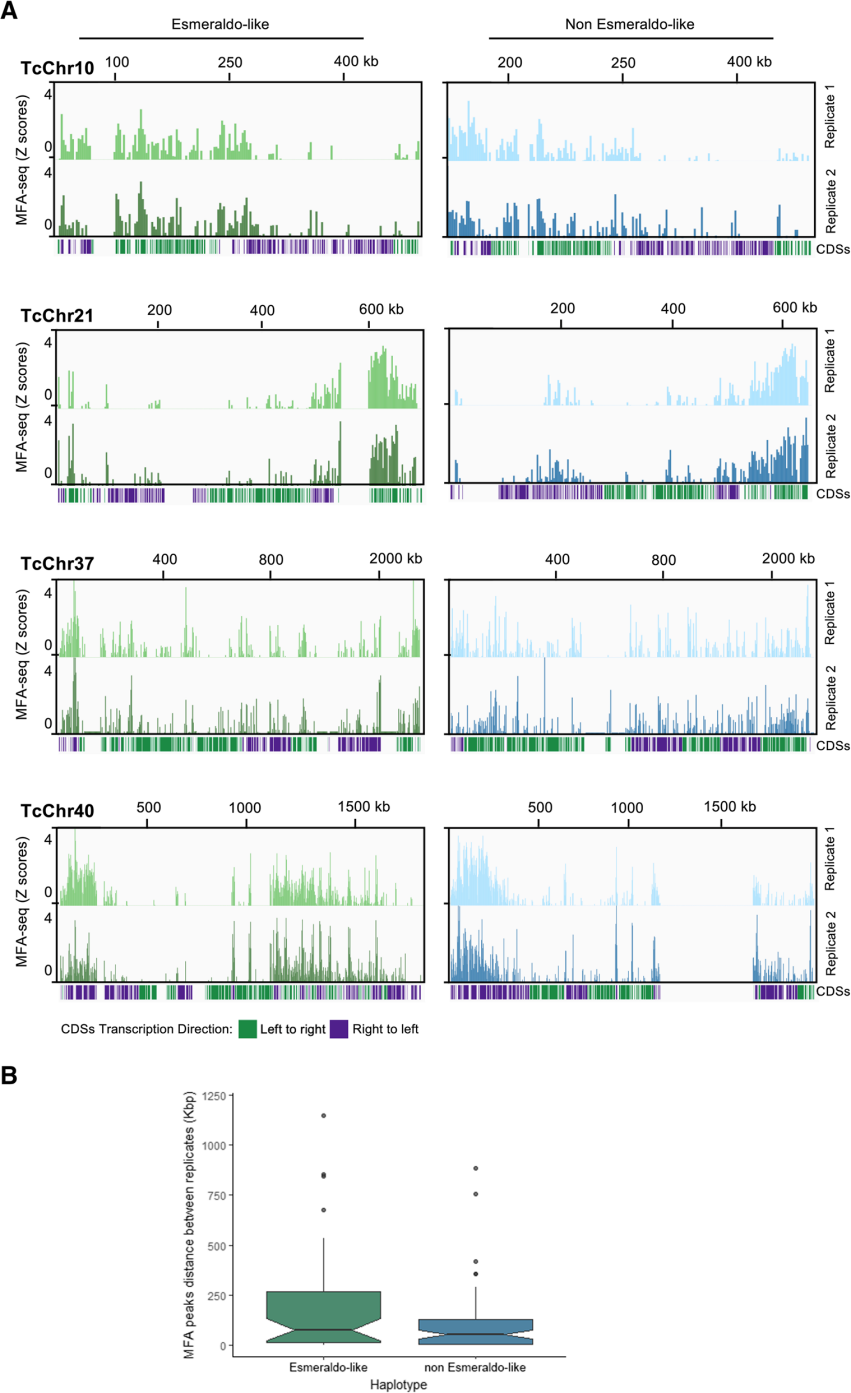
Mapping replication origins in the *T. cruzi* nuclear genome. **a**. Graphs show the extent of DNA enrichment in S phase relative to G2, in the indicated chromosomes. For each panel, the top track displays the chromosome size. The two graphs below show positive z-scores for the ratio of read depth between early S phase and G2 samples (y-axis) in a 2.5 Kbp window across the chromosome (x-axis), for two independent replicates. Finally, the track at the bottom of each panel displays the annotated transcripts. **b**. Distance (in Kbp) between the closest peak of MFA-seq replicates. 129 MFA-seq peaks pair were compared: the median distance between them was 61.3 Kbp and 19 (15%) overlaped

In order to investigate DNA replication dynamics in *T. cruzi* we analyzed the CL Brener strain, which was used for the *T. cruzi* genome sequence project [32]. Scaffolds and contigs of CL Brener strain were organized into 41 in silico chromosome pairs that vary in size ranging from 78 kb to 2.3 Mb (tritrypdb.org), although some evidences suggest that this assembly may be revised [36]. The CL Brener strain has a hybrid origin [39, 40] containing two divergent haplotypes, named Esmeraldo-like (S) and Non-Esmeraldo-like (P) [32]. We presumed that the presence of these two haplotypes would enrich our analysis since i. haplotypes are derived from different ancestors leading for evolutionary insights; and ii. findings from one haplotype could be confirmed by another, increasing robustness of our observations. We performed MFA-seq, the methodology used to infer replication origins in *T. brucei* and *L. major* [23, 26]. This technique compares the copy number of marker sequences in replicating (early-S) and nonreplicating (G2) cells. The peaks represent the early replicating sequences (origins) while later replicating sequences give rise the valleys [23]. Given the mapping complexity, consensus peaks were assigned by two different approaches, using read abundance mapped in 2500 bp. The majority of pipelines use either a null or background model or, simply, the fold change to assign a significance score to each peak region identified. Thus, exponentially growing CL Brener epimastigotes at early S (TcS) and G2 (TcG2) were sorted by flow cytometry (in two biological replicates –R1 and R2), followed by DNA extraction and deep sequencing. A total of 23,895,550 TcS (R1), 22,621,761 TcS (R2), 22,677,016 TcG2 (R1), and 21,915,604 TcG2 (R2) high quality paired-end reads were checked for quality, by sequencing adapter and contaminants removal, by quality trimming and minimum size (Table S1). The high quality paired-reads were mapped to *T. cruzi* CL Brenner, S and P  genome haplotypes, using bowtie2. The paired-end reads mapped on average of 66% to the S and 70% to the P genome haplotypes for each sample (Table S1). On average 78% of S and 87% P genomes are covered by the mapped (unique and non-duplicated) paired-end reads (Table S2). We first used the fold change to assign a significance score to each peak region identified. Two hundred forty seven putative initiation sites were predicted in the P haplotype of R1 (Table S3) and 247 of R2 (Table S4); 234 in the S haplotype of R1 (Table S5) and 235 of R2 (Table S6). Figure 1 shows the extent of DNA enrichments in S phase relative to G2 in 04 out of 41 chromosomes for both R1 and R2 replicates. Both P and S haplotypes are indicated. The visualization of data shows that peak locations are quite similar between replicates. We performed an analysis in order to compare the position of peaks between replicates. To make this analysis, we first discarded those peaks found in one chromosome in one replicate, but not in another one. Also, when we found two peaks in one replicate but only one in another replicate, we selected just the peak closer to the one of the first replicate. In the end, 129 MFA-seq peaks pair were compared (Table S7): the median distance between them was 61.3 Kbp and 19 (15%) overlapped. Note that, an average inter-origin distance was estimated as 171.1 Kbp [41].

**Fig. 2:**
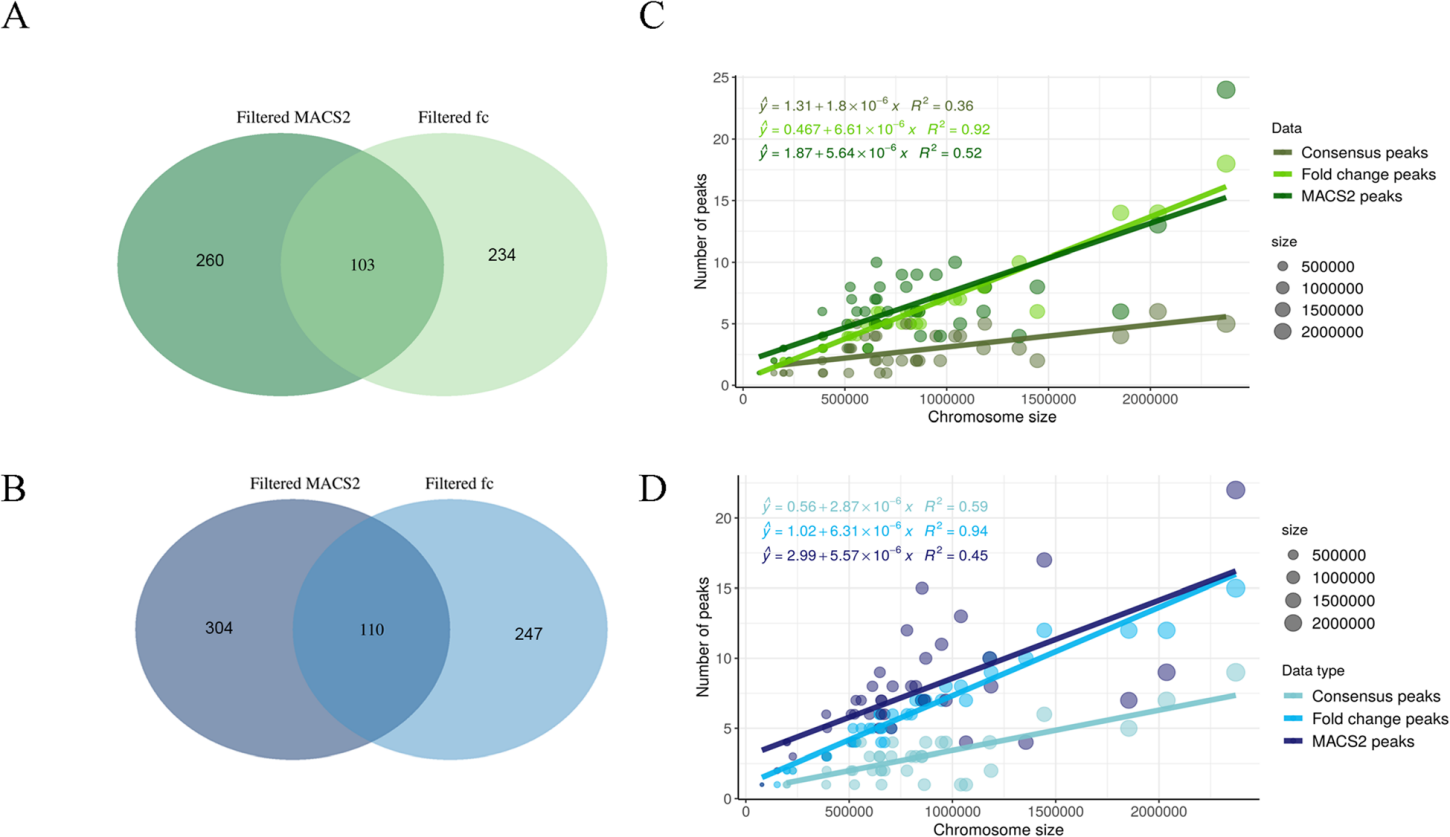
Mapping the number of replication origins in *T. cruzi* genome. Peaks determination was obtained using the MFA-seq data based on the number of reads (fold change) along the genome of cells in replicative and non-replicative phases (ratio S/G2) (fold change - fc) or determined by using the MACS2 software. The Venn diagram shows the number of peaks detected on the two CL Brener haplotypes Esmeraldo-like (**a**) and Non-Esmeraldo-like (**b**), according to the type of analysis cited above. The intersection between them corresponds to the consensus of the peaks, which have been determined as origins of replication. The number of peaks detected in all analyses was compared with the size of each chromosome in Esmeraldo like (**c**) and in Non-Esmeraldo-like (**d**). A trendline was plotted to facilitate visualization. Values of R^2^ are shown

In a second approach, peaks were obtained by MACS2 software [42]. This approach predicted 304 and 260 putative replication initiation sites, respectively, in the P haplotype R1 (Table S3) and S haplotype R1 (Table S5). Overlap between these two approaches, peaks from fold change R1 x peaks from MACS2 R1, is shown in Fig. 2a and b, with 110 and 103 consensus replication initiation sites common to the two approaches in the P (Table S8) and S haplotypes (Table S9). Consensus peaks obtained from R2 are presented on Tables S10 and S11. The number of S/G2 enriched regions increases with the size of chromosomes (Fig. 2c and d) as was observed for *T. brucei* [23] but not for *Leishmania* [26].

### Putative origins are enriched of GC content and at chromosomes peripheries in regions where transcription occurs towards the telomere

**Fig. 3:**
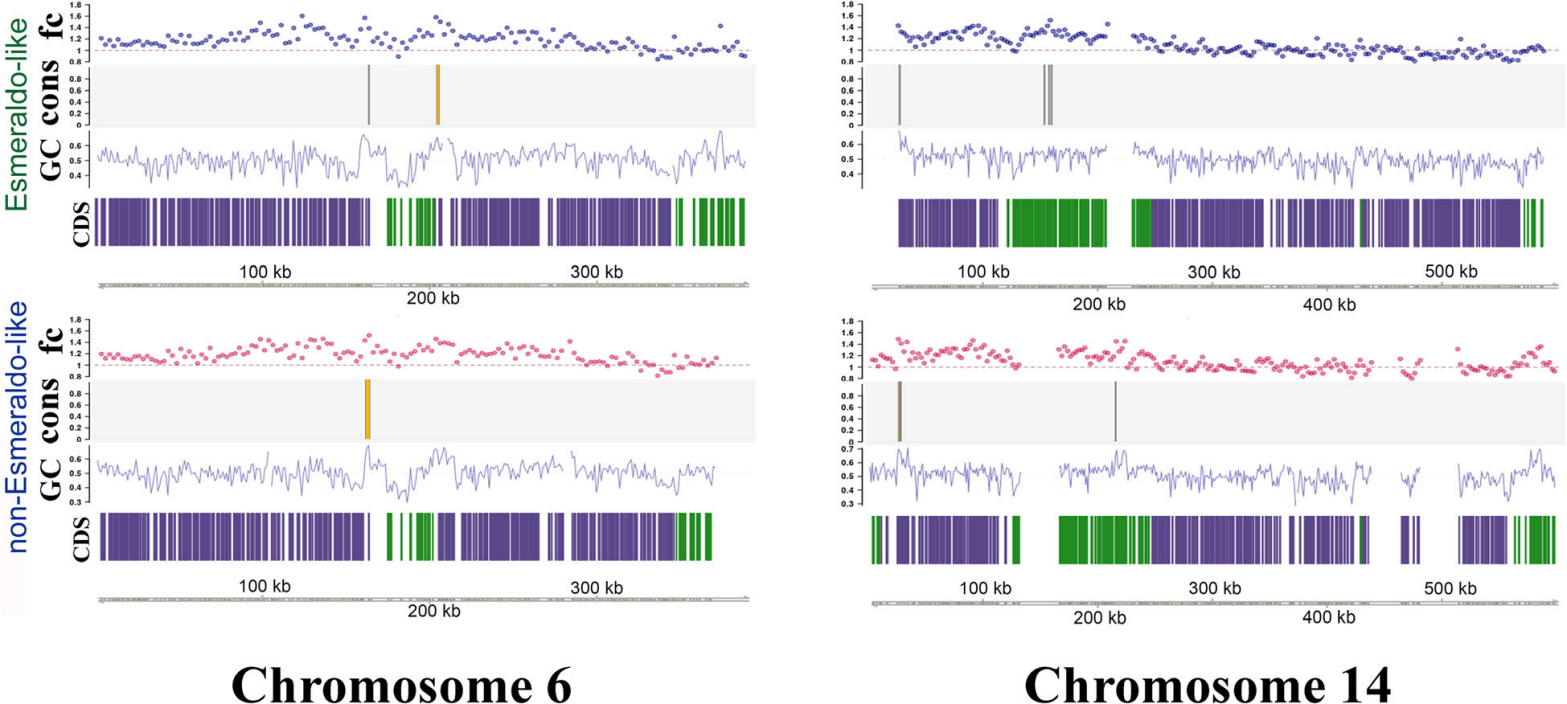
Origins location related to genomic features of *T. cruzi* chromosomes. Chromosomes 6 and 14 from S (upper panel) and P (lower panel) haplotypes were chosen as examples of this analysis. Density peaks detected at the fold change strategy (fc) and the consensus peaks (cons) were plotted to each *T. cruzi* chromosome. In the graphs also depicted the GC content (GC) along the chromosome and the directional gene clusters (CDS). Green and purple bars correspond to DGCs that are transcribed in positive and negative strand respectively

We next analyzed the genomic location of the S/G2 enriched regions, comparing the location of peaks estimated by fold change analysis, the consensus peaks (representing intersection between fold change and MACS2), GC content and transcription orientation across all *T. cruzi* chromosomes (Additional file 1 and see Fig. 3 with chromosomes 6 and 14 as examples). We noted that in chromosome 6 the ORIs were mapped in both haplotypes in a region where members of multigene families (MGF) were scarce, a “conserved compartment” according Berná et al., 2018 [43]. Whereas in chromosome 14 the ORIs were mapped at the 5′ end region enriched in members of MGF (“disrupted” compartment, according Berná et al., 2018) [43] and a second one within a “core compartment”. Therefore, we decided to analyse the presence of ORIs in the entire genome according to distribution of MGF. Although we found ORIs in both conserved and disrupted compartments, there is an enrichment of ORIs in the disrupted region (Additional file 2).

No consensus S/G2 enriched regions were predicted for chromosomes 1, 15, 20, 29 from S and 1, 2, 9 and 37 from P haplotypes. Lack of predicted origins may be due to two non-exclusive  reasons: (i) these chromosomes can be replicated with facultative origins that are not detected in MFA-seq; (ii) *T. cruzi* genome annotation presents gaps that varies between 200 bp and 20 kb. Although these gaps are not long (media of chromosomes medians are 134.6 bp in S and 144.6 bp in P), they might be compromising the identification of ORIs in some regions.

**Fig. 4:**
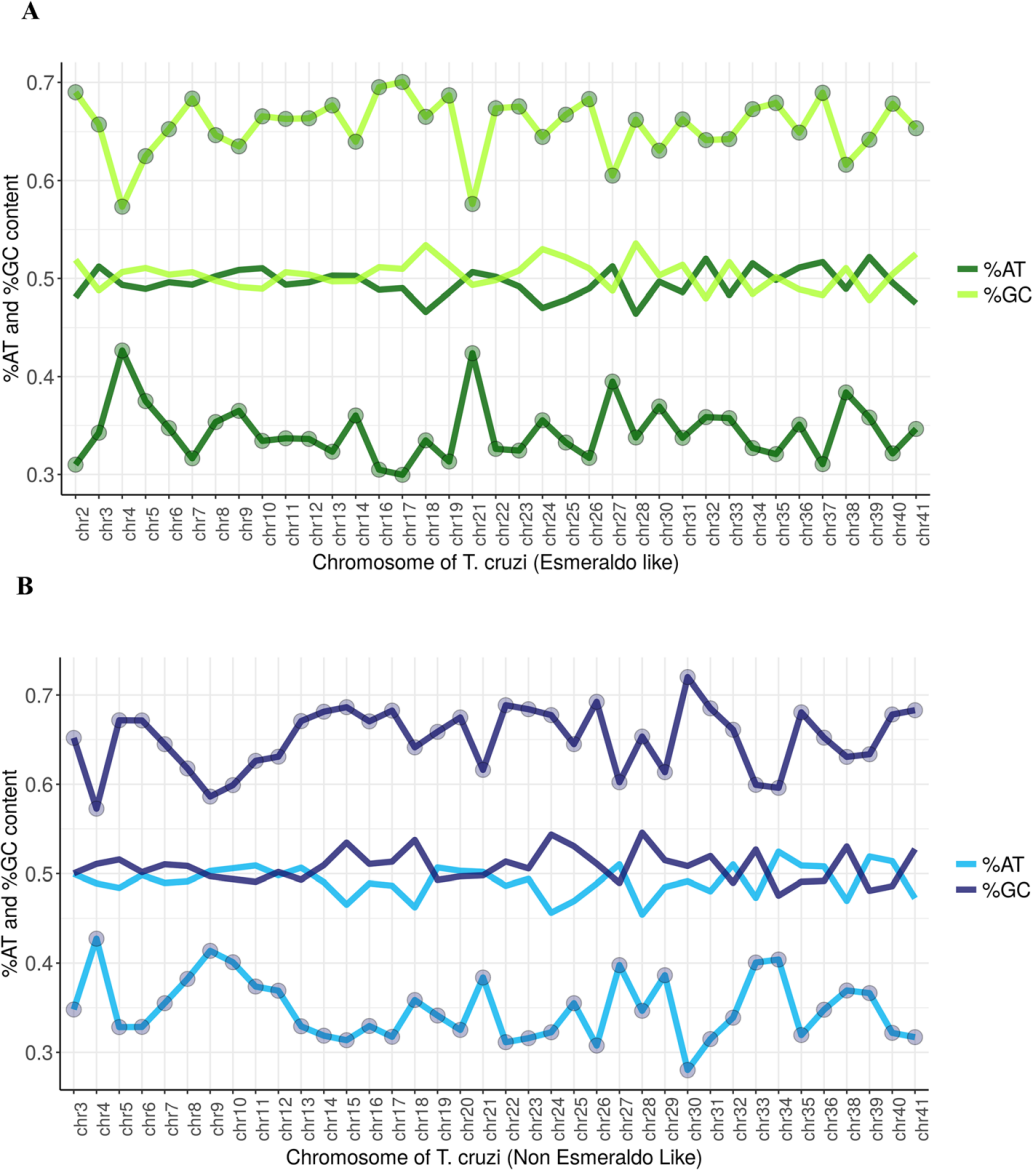
Origins are enriched of GC contents. The graphs show the percentage of AT and GC contents in all 41 chromosomes of the Esmeraldo-like (**a**) and Non-Esmeraldo-like (**b**) haplotypes. Central solid lines correspond to the contents of the AT and GC bases found in the *T. cruzi* genome. Upper and lower dotted lines correspond, respectively, to GC and AT percentages at regions designated as replication origins. When chromosomes contain more than one origin, value represents the media of CG or AT content

To better analyze GC content in consensus peaks, we next analyzed their base content in comparison to randomly selected genomic regions. The GC content throughout the random samples of binned (1083 bp) genomic regions for both P and S haplotypes are very similar: average of 40 and 44%, respectively. While the predicted ORIs were notably GC enriched (on average, 65% for both haplotypes with lower standard deviation and reaching up to 72% of GC content); the genomic regions did not exceed 54% in GC content, highlighting the larger differences for the predicted ORIs (Table 1 and Fig. 4). In addition, the CG content of DGF-1 ORIs is 68.25% (S) and 68.20% (P), while the CG content in the rest of identified ORIs is 62.36% (S) and 60.14% (P).

**Table 1:** Comparison of GC-content. Comparative of %GC-content measures, considering the genome bins of 1083 bp and the predicted ORIs. Min: %GC minimum, Max: %GC maximum, StdDev: %GC Standard deviation, Mean: %GC average estimation

GC content	Esmeraldo Like haplotype	Non-Esmeraldo Like haplotype	Predicted ORIs Esmeraldo Like	Predicted ORIs Non-Esmeraldo Like
**Average**	40%	44%	65%	65%
**StdDev**	6%	5%	4%	4%
**Min**	24%	33%	52%	53%
**Max**	53%	54%	72%	72%

**Fig. 5:**
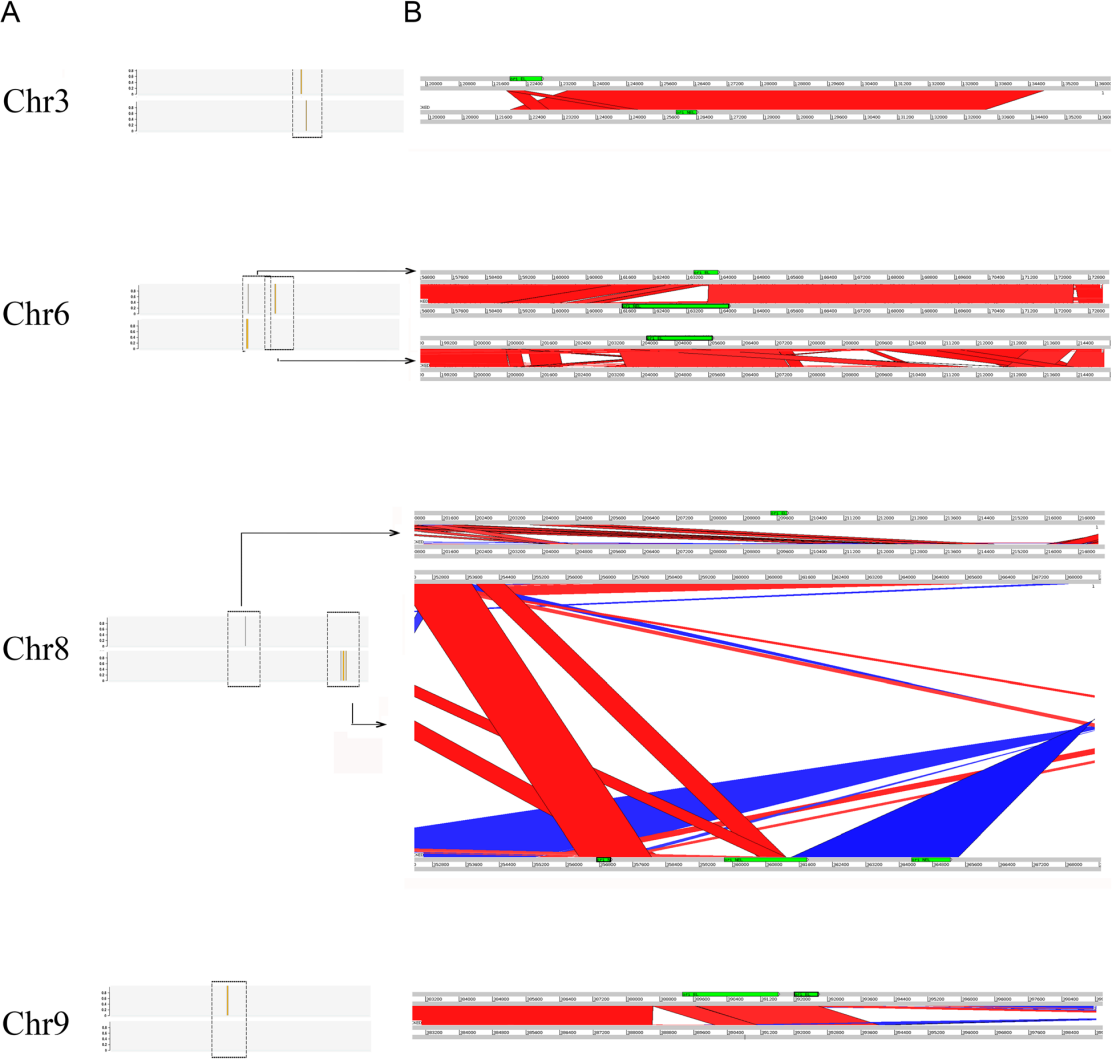
ORI-containing regions from different haplotypes are not synthenic. **a**. The ORI locations along the S (upper) and P (lower) chromosomes are shown. The box shows the region analyzed in (B). **b**. Comparisons between the S and P sequences using the Artemis Comparison Tool are shown. The green boxes indicate the ORI location, the red lines indicate syntenic regions, and the blue lines indicate inverted regions

We note in the Additional file 1 that some chromosomes seem to present putative ORI at the same location in both P and S haplotypes (see chromosomes 3, 6, 13, 16 as examples), while in others, the location changes when compared to different haplotypes. Therefore, we look for synteny on P and S haplotypes in four chromosomes as presented in Fig. 5: Chromosomes 3 and 6 that, apparently, contain ORIs in the same position in both haplotypes; chromosome 8, where putative origins were identified in both haplotypes, but in different positions; and chromosome 9, where putative ORI was identified in one haplotype but not in other (Additional file 1 and Fig. 5a). We found that syntenic regions between haplotypes do not harbor putative ORIs (Fig. 5b). This is evident on chromosome 6 where putative ORIs are in the same location, haplotypes are sythenic but the specific region that contains ORI is not synthenic. The opposite is also true. The putative ORI is found in a synthenic region on Chr3-P and this region does not contain ORI in Chr3-S. Therefore, it seems that different position of ORIs is not due to chromosomes rearrangements. However, since even in the syntenic regions of S and P haplotypes there are differences between the homologous chromosomes due to the duplication/deletion events occurred during *T. cruzi* evolution, we need further studies in order to understand the molecular bases of origins location in this parasite.

**Fig. 6:**
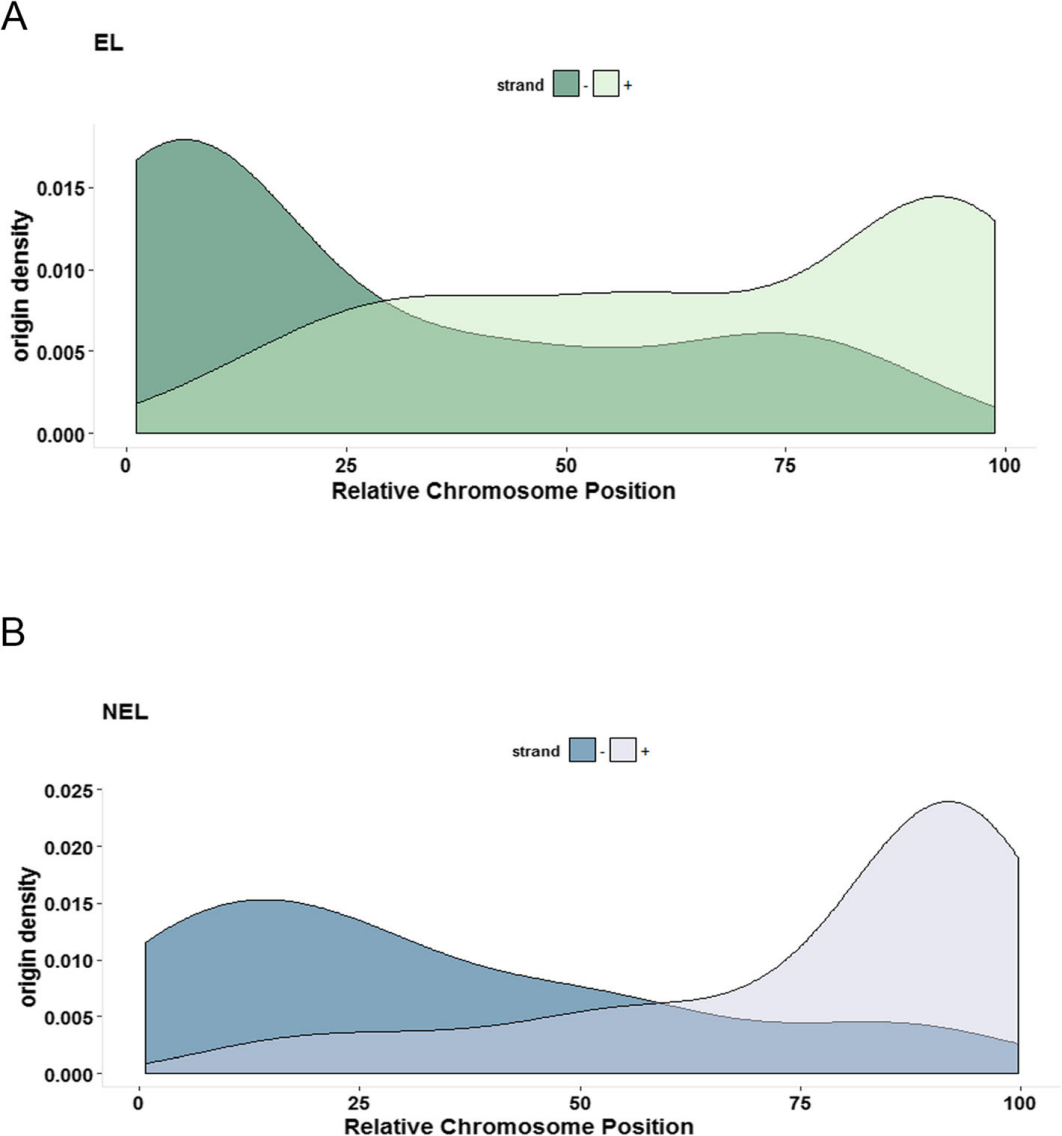
Origins are enriched at chromosomes ends. The ORI density was plotted to all 41 *T. cruzi* chromosomes according to their relative chromosome position, which was normalized from 0 to 100 as start and end of each chromosome. (**a**) Origins density and transcription orientation relative to chromosome location in S haplotype. The y-axis in a density plot is the probability density function for the kernel density estimation. (**b**) Overall gene density and transcription orientation along each chromosome in P haplotype. (−) Genes transcribed in the negative strand; (+) genes transcribed in the positive strand

Next, we analyzed the ORI location across the genome and we observed that the origins are preferentially located at the edges of chromosomes (Fig. 6). After that, we asked how transcription orientation on regions is containing putative origins. So, we classified DGCs whether the corresponding coding DNA region came from positive and negative strands. Strikingly, there was a clear correlation between replication initiation site location (beginning or end of chromosome as annotated in the trytripdb.org) and transcription orientation: more replication initiation sites were located at the beginning of chromosomes in regions where transcription is also orientated towards the chromosomes’ beginning (negative strand, Fig. 6); and, likewise, more initiation sites were predicted at the end of chromosomes, where the orientation of transcription is toward the end of chromosome (positive strand, Fig. 6). We also analyzed overlapped peaks between R1 and R2 and those peaks are indeed preferentially located at the chromosomes edges (Additional file 3).

### Most putative origins are located at dispersed gene family 1 (DGF-1) genes

To further investigate the predicted replication initiation sites in the *T. cruzi* genome, we examined what genomic features they correlate with. We classified the *T. cruzi* genome into CDS, transcribed inter-CDS, divergent SSR and convergent SSR sequences and asked about their colocalization with the predicted consensus replication initiation sites (Additional file 4). We found that in P haplotype, four putative origins were predicted in divergent SSRs, three in convergent SSRs, seven in inter-CDS regions and 96 within CDS. Remarkably, among the 96 putative origins in CDS at P haplotype, 71 (64.5%) were located in DGF-1 family genes (Table S8). In S haplotype, three putative origins were in divergent SSRs, one in a convergent SSR, three in inter-CDS regions, and 96 in CDSs. Like in the P haplotype, most of the putative CDS origins (68, or 66% of the total) were also located in DGF-1 family genes in S haplotype (Table S9). To better understand these origins containing DGF-1, we performed three analysis as present in Additional file 5. We could see that ORIs were found at longer DGF-1, most of them in DGF-1 genes (and not at pseudogenes), and in DGF-1 tandem arrays. In addition, since we didn’t identify ORIs at Chr1, Chr2, and Chr 37 from S haplotype and from Chr1, Chr15, Chr20 and Chr29 from P haplotype, we asked if it could be due to lack of DGF-1 genes in these chromosomes. However, four of them contain DGF-1 genes (Chr1S- 1 DGF-1 gene; Chr 2S – 1 gene, Chr 1P – 1 gene, and Chr20P – 3 genes) and therefore, lack of origin might not be explained by lack of DGF-1 genes in these chromosomes. We also analyzed where ORIs are localized in those chromosomes that do not harbor DGF-1 genes. Table S10 shows CDSs containing ORIs in these chromosomes. Concerning R2, in –P haplotype 12/86 origins are in inter-CDS, 4/86 within convergent SSR, and 69/86 within CDS (Table S11), while in S haplotype 9/78 ORI are in inter-CDS, 1/78 is within convergent SSR, and 68/78 within CDS (Table S12). We also checked if putative ORIs were at DGF-1 genes in replicate 2. We found that 22% of putative ORIs were localized in DGF-1 in P haplotype and 27% of them in DGF-1 in S haplotype. The enrichment of ORIs at DGF-1 genes is also true for peaks that are overlapped between R1 and R2–45% of CDS peaks are in DGF-1 in S haplotype and 27% in P haplotype – Additional file 3 and Table S13. Since DGF-1 genes represent only 3.33% of the total genome in base pairs on both haplotypes (Table S14) and are not the most abundant multigene family in *T. cruzi* (Additional file 6), we conclude there is a great enrichment of replication initiation sites at DGF-1 genes.

Since *T. cruzi* in silico chromosomes are incomplete, we double-checked the presence of ORIs at chromosome ends analyzing regions previously mapped as chromosomes extremities [36]. Nineteen putative origins in the P haplotype and 19 in S (Table S15) matched with previously annotated subtelomeric regions. Considering that the 38 subtelomeric regions ORI positive represent less than 0.2% of genome in size, and 17.8% of putative origins were located at these regions, we conclude that replication initiation is enriched at subtelomeric regions in *T. cruzi*. All putative origins found in subtelomeric regions were inside DGF-1 genes, meaning that 21 and 19% of DGF-1 associated putative origins from the P and S haplotypes, respectively, are at the subtelomeric region.

### Putative *T. cruzi* origins locations are associated with increased sequence variability

**Fig. 7:**
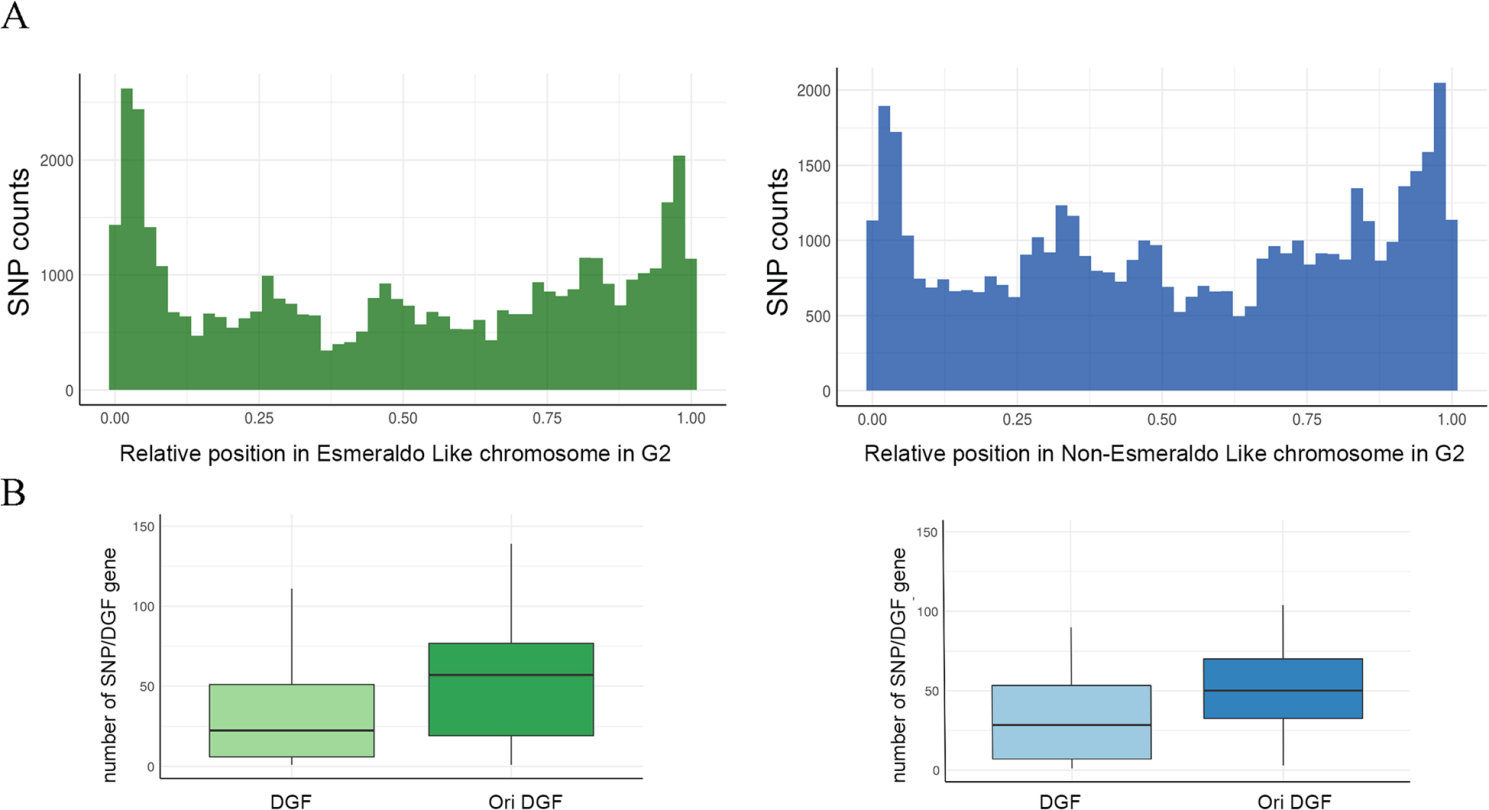
Single nucleotide polymorphisms (SNPs) are enriched in origins containing regions. (**a**) SNPs distribution at chromosomes regions in Esmeraldo-like (Green bars) and Non Esmeraldo-like (blue bars) haplotypes genome. Chromossomes sizes were normalized to 1 and SNPs positions were mapped accordingly. (**b**) Number of SNPs per DGF-1 gene that contains (DGF-1 Ori) or not (DGF-1) origins inside their CDS in Esmerado-like (Green box) and Non Esmeraldo-like (Blue box). Wilcoxon signed-rank test yielded a *p*-value of 0.0003273 for S haplotype and of 0.0003162 for P haplotype

Subsequently, we asked if chromosome peripheries and DGF-1 genes containing putative origins are associated with signatures of sequence variability. To address this, we first analyzed single nucleic polymorphism (SNP) across the entire genome. To this end, mapped reads from MFA-seq were compared and SNPs were detected. In performing this analysis, we found that the frequency of SNPs was highest towards the periphery of the chromosomes in both haplotypes (Fig. 7a). Next, we compared the frequency of SNPs in DGF-1 genes predicted to contain putative origins with those without predicted origins: SNPs were much abundant in the former (Fig. 7b), indicating an association between sites of replication initiation and genetic variability strongly corroborating to genetic variability.

## Discussion

Here we investigated the DNA replication characteristics using Marker Frequency Analysis. The number of putative ORIs obtained either by fold change or by MACS2 is higher than obtained after prediction of the consensus peaks suggesting that using the consensus approach might have a more reliable set by excluding false positive ORIs. However, this strategy may also exclude predicted origins that could be relevant for our analysis. Given that the number of *T. cruzi* S/G2 enriched regions predicted by both strategies exceeds the number of predicted origins in both *T. brucei* and two species of *Leishmania* [23, 26], we decided to be more stringent and continue the follow analysis by using the putative ORIs obtained by the consensus prediction approach. In order to characterize the ORIs in *T. cruzi* genome, we first looked for a consensus sequence in ORIs but we dind’t find. Also, we extracted ORIs presented at both R1 and R2 and mapped them via BLASTn against *Leishmania major, L. mexicana,* and *T. brucei* genomes. However, we dind’t get any hit. Then, we looked for the predominant content at these putative replication sites. It was possible to observe that the majority of predicted origins in *T. cruzi* are GC enriched regions. This data reinforces the fact that S/G2 enriched regions could act as origins since some primary sequence elements have been widely associated with the replication origins in various organisms. In *S. cerevisiae*, origins are enriched with AT- sequences but in the other eukaryotes, origins are GC-rich sequences [44], including CpG islands and G-rich elements of the Origin G-rich repeated element (OGRE), which have great potential to form secondary DNA conformations, such as G-quadruplex [45, 46], and intercalated motif (i-motif) [47, 48].

To get an overview of DNA replication initiation location across the genome, we also determined origin density in relation to chromosome length. We could clearly see that origins are constrained at chromosomes periphery suggesting greater abundance with telomere proximity. This finding indicates that one replication fork drives towards the chromosome end, meaning it provides a relatively less important contribution to chromosome replication. Therefore, we wondered if the putative ORIs detected would be enough to allow *T. cruzi* to fully replicate its chromosomes during S phase. Previous analysis by DNA combing (which can detect any replication initiation event, including constitutive, flexible and dormant origins, but without reference to genome location) in *T. cruzi* CL Brener suggested a median inter-origin distance of 171.1 kb [41], which can be extrapolated to a total of 85 origins, which is close to 103 and 110 putative consensus origins mapped in the P and S haplotypes in this analysis. Therefore, it indicates that MFA-seq analysis was able to cover origins used by *T. cruzi* to replicate entire genome at least in an unstressed condition.

Following the characterization of *T. cruzi* ORIs, we investigated the origins position at the edges of chromosomes and correlated them with the transcription profile at that region. In fact, the orientation of transcription towards chromosomes telomeres is a feature of the *T. cruzi* genome (Additional file 7), suggesting the greater abundance of putative origins in subtelomeres generates head-on transcription-replication collisions as the replisomes move towards the centers of the chromosomes. In accordance with this scenario is the fact that *T. cruzi* subtelomeric regions are indeed transcribed since RNA-seq data detected RNAs originated from this location (www.tritrypdb.org). This is an intriguing result because prokaryotes and eukaryotes rely on some resources to avoid conflicts between transcription and replication machineries, such as temporal and special separation of transcription and replication processes, avoid stalled RNA polymerases in the genome, and orientation of highly transcribed genes in the same direction of replication fork movement [49]. Even organisms that present genes organized in operons (in the case of *C. elegans*) or in polycistron (as *T. brucei*), origins are located in transcription start sites warranting the replication fork movement in the same direction than transcription [23, 50]. Therefore, it tempts us to propose that in *T. cruzi*, sites of replication initiation seem to be strategically positioned to favor replicative stress and, consequently, to promote recombination events observed at *T. cruzi* subtelomeric regions [36–38]. In other words, subtelomeric replication initiation, where transcription occurs towards chromosome ends, may be at least one of the sources of DNA breaks, because transcription has been shown to arrest replication fork progression [51, 52]. Stalled replication forks can accumulates ssDNAs and become prone to DSBs [53] that is repaired by homologous recombination [54]. Besides the favoring of DSB by stalled replication fork due its collision with transcription machinery, stalled replication fork can promote the action of translesion DNA polymerases [14], which can catalyze error-prone DNA synthesis [15] or can accumulate ssDNAs and become prone to DSBs that may be repaired by microhomology end joining, whose consequences include base pair substitution [55].

Interestingly, the majority of ORIs identified in this analysis was preferentially positioned inside the CDSs, mainly in the DGF-1gene. It is known that many members of the DGF-1 gene family are located in subtelomeric regions, where they may be prone to variability [56]. In order to ask if the putative origins within the DGF-1 genes are also located in subtelomeric regions, we further mapped them within the 49 subtelomeric regions described by Moraes Barros et al., 2012, who detailed the organization and gene content of *T. cruzi* chromosomes ends [36], and confirmed that all putative origins identified in that region were inside the DGF-1 gene. It has been argued that trypanosomatids limit the presence of ORC-defined origins to SSRs, leading to widely spaced origins, in order to limit binding of the initiator and thereby limiting transcription-replication clashes in the context of multigenic transcription [23, 26, 57]. Thus, the presence of putative origins within CDS, and in particular within DGF-1 genes, in *T. cruzi* is striking. Data from mammalian cells showed that replication forks from origins inside genes are prone to collapse due to collision between transcription and replication machinery, which triggers DSB formation and chromosomal rearrangement [58]. We envisage a similar event could occur in the replicative *T. cruzi* epimastigote forms examined here: replication from DGF-1 gene origins has evolved to favor collision with transcription during S phase since it favors genetic variability. Further studies are necessary to validate this hypothesis. We need to explore, for instance, whether (and which) DGF-1 is transcribed at S phase of replicative forms and if these DGF-1 transcribed at replicative forms would be expressed in the infective forms where the larger repertoire of DGF-1 isoforms could contribute for infection. In fact, it has already been shown that trypomastigotes [59] express DGF-1, and some members of this protein are differentially expressed during the life cycle stages [60], though additional analysis of this family’s expression during the *T. cruzi* life cycle needs to be conducted.

## Conclusions

In conclusion, this paper provides the first attempt to map sites of replication initiation across the uniquely challenging *T. cruzi* genome. Though we cannot be certain the regions of increased S/G2 enrichment truly represent origins, since we have not mapped localization of the ORC machinery, our data suggest that while some *T. cruzi* origins are located in non-transcribed regions as well as those seen in *T. brucei* and *Leishmania*, many more appear strategically localized to produce genetic variability at the chromosomes periphery, with a strong focus on DGF1-genes. Whether this is because of replication-transcription conflicts derived from DNA forks emanating from ORC-defined origins in these loci, or if we have detected replication that may arise from transcription, as proposed in *Leishmania* [29] requires further analysis. Further work is also warranted to ask what features of the *T. cruzi* DGF-1 genes dictate DNA replication initiation, and why variability in these loci might be needed in order to guarantee the success of *T. cruzi* infection.

## Methods

### Parasites

*Trypanosoma cruzi* CL Brener clone was obtained from CL *Trypanosoma cruzi* strain isolated from a *Triatoma infestans* collected in Rio Grande do Sul, Encruzilhada, South Brazil, in1963 [61, 62]. Epimastigote forms were maintained in Liver Infusion Tryptose (LIT) at 28 °C, at a density of 3 × 10^6^ parasites.ml^− 1^.

### Cell sorting

Epimastigotes in exponential growth (about 1x10^9^parasites total) were centrifuged at 1258 g for 5 min, washed in PBS and incubated with a propidium iodide (PI) solution (3.4 mM Tris-HCl ph 7.4; 0.1% NP-40; 700 U/L RNAse; 10 mM NaCl; 0.075 mM propidium iodide – PI) for 10 min. After the incubation time, the parasites were sorted in early S and G2/M phases using a FACSAria II (BD Biosciences) at the Rede de Plataformas Tecnológicas (RPT-FACS), FIOCRUZ - PR. The parasites in early S or G2/M were collected during the sorting in lysis buffer (1 M NaCl; 10 mM EDTA; 50 mM Tris-HCl pH 8.0; 0.5% SDS; 0.4 mg/ml Proteinase K; 0.8 μg/ml Glycogen) and then, they were incubated for 2 h at 55 °C. The lysate was stored at − 20 °C until the DNA extraction. Genomic DNA from early S and from G2/M was extracted according to manufactures instructions, using the QIAmp DNA Micro Kit (Qiagen) and quantified by NanoDrop 2000 UV-Vis (Thermo Scientific).

### MFA-seq analysis

The DNA from early S and G2/M phases were analyzed by Marker Frequency Analysis (MFA-seq). Firstly, the samples were prepared to library and sequencing Illumina at the Polyomics Glasgow Center (University of Glasgow, Scotland) and the paired-reads obtained for each sample were pre-processed by an in-house pipeline for exclude contaminants from Phix using Bowtie2 [63], filtering and trimming low quality reads, using the Trimmomatic (version 0.36, parameters: ILLUMINACLIP:NextGenPrimerAdaptersUniVec.fa:2:30:10, LEADING:5, TRAILING:5, SLIDINGWINDOW:15:25, MINLEN:35, HEADCROP:0) to exclude vector, adapter and index sequences. The paired-reads were aligned to the reference genome of *T. cruzi*, CL Brener strain (TriTrypDB release 32, version 2015-12-07**;** CLBrenerNonEsmeraldo-like: https://tritrypdb.org/common/downloads/release-32/TcruziCLBrenerNon-Esmeraldo-like/fasta/data/TriTrypDB-32_TcruziCLBrenerNon-Esmeraldo-like_Genome.fasta, and CLBrenerEsmeraldo-like https://tritrypdb.org/common/downloads/release-32/TcruziCLBrenerEsmeraldo-like/fasta/data/TriTrypDB-32_TcruziCLBrenerEsmeraldo-like_Genome.fasta). The pipeline for origin (ORI) regions prediction was composed by four main steps: (i) getting the fold change between S/G2 phases and calculating the coverage values in fixed bins of 2.5 kb regions along each chromosome; S/G2 enrichment by fold change analysis were classified by the arbitrarily percentile rank of 2% most significant with FDR lower than 0.05. (ii) estimating the fold change and enriched regions using the MACS2 software (version 2.1.1) [64]; S/G2 enrichment were defined as those genomic regions whose S/G2 fold changes were classified by the arbitrarily percentile rank of 5% most significant with FDR lower than 0.05. (iii) applying a quality threshold over the coverage estimation and (iv) obtaining the consensus region between the first two steps. The S and G2 phase paired-reads were aligned against the genomic sequences of the Esmeraldo-like and non Esmeraldo-like haplotypes using the software Bowtie2 v 2.2.9 [65], with the parameters “end-to-end” whole paired-read alignment (−-very-sensitive), reporting only the best alignment (−k 1) for paired-reads with multiple alignments; the mixed (−-no-mixed) and discordant (−-no-discordant) alignments were discarded followed by the subsequent elimination of duplicate reads using the samtools program ‘rmdup’. The .bam alignment files were converted into .bed (using the tool ‘bedtools bamtobed’) format for later analyses. Using the genomic fasta file, each chromosome were split out in fixed windows of 2.5Kb size using the ‘bedtools makewindow’ function from bedtools v2.27.1 package [66], then were calculated the number of reads mapped in each one using ‘bedtools coverage’ function. This process was conducted individually for each sample result. Finally, the coverage values were normalized by the total of reads mapped in each condition and also to estimate the ratio between S and G2 phases (fold change). Similarly, were used S and G2 data to estimate peaks of enriched regions in the genome using ‘macs2 callpeak’ function from MACS2 software, which uses a Poisson distribution to calculates a dynamic Poisson parameters for each region to obtain a distribution having more flexibility than the negative binomial distribution [64]. In both fold change results was applied a threshold on the percentile of 0.98 for the S/G2 fold change and 0.95 for the MACS2 predicted peaks. For MACS2 was also applied a threshold on the q-value lower than 0.01. The peaks above the thresholds were selected as enriched regions. Finally, the genomic coordinates of both approaches (fold change and MACS2) were crossed in order to establish a consensus of enriched regions denominated as ORIs. Using the ‘bedtools intersect’ function, the regions overlapping in at least one base were elected as significantly enriched regions between S and G2. Subsequently, the data were integrated with other genomic information such as %AT-GC contents, UTR regions, CDS, genes, transcripts, etc. Data integration was performed through a joint plot of experimental and genomic information using the Gviz version 1.20.0 package [67] and the in house webtool (Inada et al., 2018, unpublished).. The datasets generated and/or analyzed during the current study are available in the NCBI BioProject repository, under the accession code PRJNA635749 (MFA-seq bioproject) and BioSample codes SAMN15052360 (Epimastigote at G2 stage, replicate 1), SAMN15052361 (Epimastigote at G2 stage, replicate 2), SAMN15052345 (Epimastigote at S stage, replicate 1), and SAMN15052356 (Epimastigote at S stage, replicate 2) (see Table 2 for details).

**Table 2:** The NCBI Accessions for the BioProject, BioSamples and respective raw data files related to MFA-Seq analysis

	Description	Accession
**BioProject**	Trypanosoma cruzi Epimastigote MFA-seq	PRJNA635749
**BioSample**	Epimastigote G2-phase Replicate 1	SAMN15052360
**Experiment**	Epimastigote G2-phase R1	SRX8421487
**Rawdata**	Tcruzi_G2phaseReplicate1_R1.fastq.bz2	SRR11871783
**BioSample**	Epimastigote G2-phase Replicate 2	SAMN15052361
**Experiment**	Epimastigote G2-phase R2	SRX8421488
**Rawdata**	Tcruzi_G2phaseReplicate2_R1.fastq.bz2	SRR11871782
**BioSample**	Epimastigote S-phase Replicate 1	SAMN15052345
**Experiment**	Epimastigote S-phase R1	SRX8421489
**Rawdata**	Tcruzi_SphaseReplicate1_R1.fastq.bz2	SRR11871781
**BioSample**	Epimastigote S-phase Replicate 2 *T. cruzi*	SAMN15052356
**Experiment**	Epimastigote S-phase R2	SRX8421490
**Rawdata**	Tcruzi_SphaseReplicate2_R1.fastq.bz2	SRR11871780

### GC content estimation

The predicted ORI consensus regions were used to estimate the GC-content and presented an average size of 1083 bp. Each one of the chromosomes from P and S haplotypes were binned into 1083 bp sections, and the average value across each bin was used to calculate the mean and the standard deviation of GC-content for each chromosome, considering the whole chromosome. To compare the GC-content between predicted ORIs and genomic region, we took 30,000 random samples, ranging from 1 up to 30 bins for each chromosome, in accordance to the maximum predicted ORIs in one chromosome by MACS2 software. The genomic regions with zero GC-content were removed from the samples. The mean and standard deviation of GC-content were estimated for all bins in each chromosome, and further for each sample, them a final estimation for each genome was calculated between all samples.

### Detection of single nucleotide polymorphism (SNP)

The analyses and parameters were defined and conducted as previously described [68]. Briefly, the sequencing data were aligned using the BWA v0.7.12-r1039 program [69]. After the alignment, the SNPs were predicted using the GATK v3.7 tool (Genome Analysis Toolkit) [68], based on the best practices and standard protocol (https://software.broadinstitute.org/gatk/best-practices/). The predicted SNPs, along the entire genome of *T. cruzi*, were subsequently submitted to a quality control filter (60.0, MQ > 40.0 - the Root Mean Square of the mapping quality of the reads across all samples; MQRankSum > − 12.5 - An u-based z-approximation from the Mann-Whitney Rank Sum Test for mapping qualities; ReadPosRankSum > − 8.0 - the u-based z-approximation from the Mann-Whitney Rank Sum Test for the distance from the end of the read for reads with the alternate allele).

### Analysis of syntheny

Syntenic regions between P and S chromosomes were identified using BLASTn [70] alignments (E-value: 1E-05), followed by visualization in Artemis Comparison Tool (ACT) [71]. Annotations of chromosome replication origin regions were added based on Table S8 and S9.

## Supplementary information

**Additional file 1. **Origins location related to genomic features of *T. cruzi* chromosomes. Chromosomes from S (upper panel) and P (lower panel) haplotypes are presented. Density peaks detected at the fold change strategy (a) and the consensus peaks (b) were plotted to each *T. cruzi* chromosome. In the graphs also depicted the GC content (c) along the chromosome and the directional gene clusters (d). Green and purple bars correspond to DGCs that are transcribed in positive and negative strand respectively.

**Additional file 2. **Distribution of consensus replication origin regions located at DGF-1 genes according to their location at conserved (C- top) or disrupted (C-intermediate) *T. cruzi* genome domains (according to (43)). Both (B-below) represents DGF-1 containing origins that could be in either one domain. B. Examples of replication origins domains at conserved, disrupted and in between both domains. According to (43), DGF-1, RHS and GP63 (orange) may be located either at disrupted (red) or conserved (blue) compartments. The *T. cruzi* genome was loaded at UCSC genome browser and these 3 gene groups were colored in accordance. ORIs genome coordenates are shown in blue.

**Additional file 3.** Analysis of ORIs found at both R1 and R2 replicates. A. Consensus origin regions from replicates 1 (R1) and 2 (R2) were considered overlaped if they share at least one nucleotide. 60% (15/25) and 27% (3/11) of overlapped origins were located at DGF-1 genes in Esmeraldo-like and Non Esmeraldo like haplotype. B. Origins that were mapped at CDS were evaluated according their chromosome location.

**Additional file 4.** Schematic representation of a *T. cruzi *genomic region. CDSs are represented in green or purple vertical lines. Clusters of CDSs that are transcribed in the same direction represent a DGC. Arrows indicate the orientation of transcription. Green arrow is the transcription orientation of genes transcribed in positive strand (green) or in negative one (purple).

**Additional file 5. **Distribution of ORIs among DGF-1 genes. A. Scatter plot of DGF-1 genes distribution according their gene size (in bp). Total DGF-1 and DGF-1 containing replication origins were compared regarding their gene size. *P-value < 0.005 – unpaired Welch  T-test. B. Presence of ORIs in DGF-1 genes or pseudogenes. C. Presence of ORIs in DGF-1 genes/pseudogenes within DGFF-1 in tandemly arrays or at isolated DGF-1.

**Additional file 6. **Percentage of genes (in base pairs) in *T. cruzi* genome. The percentage of indicated genes belonging to the multigene family is represented for Sand P haplotypes. Sizes of genes in base pairs were normalized to the genome size.

**Additional file 7.** Transcription orientation along chromosomes. Overall gene density and transcription orientation along each chromosome in S haplotype (A) and P haplotype (−) Genes transcribed in the negative strand; (+) genes transcribed in the positive strand.

**Additional file 8: Table S1.** Total number of raw paired-end reads for each sample and respective replicate, followed by the high quality paired-end reads after the pre-processing quality approach and the percentage of mapped paired-end reads in the CL Brenner EL and NEL genome haplotypes. **Table S2.** The average of %Genome coverage based on paired-end reads for each sample mapped to the genome haplotypes, based on the paired-end reads with highest mapping quality. **Table S3.** Filtered fold change analysis and MACS2 of MFA-seq in non-Esmeraldo-like haplotype **Table S4.** Filtered fold change analysis and MACS2 of MFA-seq in non-Esmeraldo-like haplotype (replicate 2) **Table S5.** Filtered fold change analysis and MACS2 of MFA-seq in Esmeraldo-like haplotype **Table S6.** Filtered fold change analysis and MACS2 of MFA-seq in Esmeraldo-like haplotype (replicate 2) **Table S7.** Comparison between MFA-seq peaks detected at replicates 1 and 2 **Table S8.** Position of MFA-seq peaks (Consensus) in non-Esmeraldo-like haplotype **Table S9.** Position of MFA-seq peaks (Consensus) in Esmeraldo-like haplotype **Table S10.** Genes where ORIs where found in chromosomes that do not harbor DGF-1 **Table S11.** Position of MFA-seq peaks (Consensus) in non Esmeraldo-like haplotype (replicate 2) **Table S12.** Position of MFA-seq peaks (Consensus) in Esmeraldo-like haplotype (replicate 2) **Table S13.** MFA-seq peaks (Consensus) that overlap between replicates **Table S14.** Percentage of DGF-1(bp) in the entire genome **Table S15.** Genomic location and IDs of subtelomeric genes described at Moraes-Barros 2012

## Data Availability

The reference genome used in the present work is available at TriTrypDB release 32, version 2015-12-07 (CLBrenerNonEsmeraldo-like: https://tritrypdb.org/common/downloads/release-32/TcruziCLBrenerNon-Esmeraldo-like/fasta/data/TriTrypDB-32_TcruziCLBrenerNon-Esmeraldo-like_Genome.fasta, and CLBrenerEsmeraldo-like https://tritrypdb.org/common/downloads/release-32/TcruziCLBrenerEsmeraldo-like/fasta/data/TriTrypDB-32_TcruziCLBrenerEsmeraldo-like_Genome.fasta). The datasets generated and/or analyzed during the current study are available in the NCBI BioProject repository, under the accession code PRJNA635749 and BioSample codes SAMN15052360, SAMN15052361, SAMN15052345, SAMN15052356 (see Table 2).

## Abbreviations

S: Esmeraldo-like
P: Non-Esmeraldo-like
ORI: Replication origin
MFA-seq: Marker frequency analysis
ORC: Origin recognition complex
DSB: Double strand break
TS: Trans-sialidase
CDS: Coding DNA sequence
DGC: Directional gene cluster
SSR: Strand switch region
DGF-1: Dispersed gene family 1
SNP: Single Nucleotide Polymorphism
ACT: Artemis Comparison Tool
