# Supplementary material for: Replication origin location might contribute to genetic variability in Trypanosoma cruzi
Source: BMC Genomics. 2020 Jun 22;21:414. doi: 10.1186/s12864-020-06803-8 (PMC7310030; doi:10.1186/s12864-020-06803-8)
Supplement: Supplementary file 1 — Additional file 1. Origins location related to genomic features of T. cruzi chromosomes. Chromosomes from S (upper panel) and P (lower panel) haplotypes are presented. Density peaks detected at the fold change strategy (a) and the consensus peaks (b) were plotted to each T. cruzi chromosome. In the graphs also depicted the GC content (c) along the chromosome and the directional gene clusters (d). Green and purple bars correspond to DGCs that are transcribed in positive and negative strand respectively. [file 12864_2020_6803_MOESM1_ESM.docx]

**Additional file 1**. Origin location related to the genomic features of *T. cruzi* chromosomes. Chromosomes from the EL (upper panel) and NEL (lower panel) haplotypes are presented. The density peaks detected by the fold change strategy (a) and at the consensus peaks (b) were plotted on each *T. cruzi* chromosome. The graphs also depict the GC content (c) along the chromosome and the directional gene clusters (d). The green and purple bars correspond to DGCs that are transcribed from the positive and negative strands, respectively.
