# Supplementary material for: Replication origin location might contribute to genetic variability in Trypanosoma cruzi
Source: BMC Genomics. 2020 Jun 22;21:414. doi: 10.1186/s12864-020-06803-8 (PMC7310030; doi:10.1186/s12864-020-06803-8)
Supplement: Supplementary file 2 [file 12864_2020_6803_MOESM2_ESM.docx]

Additional file 2

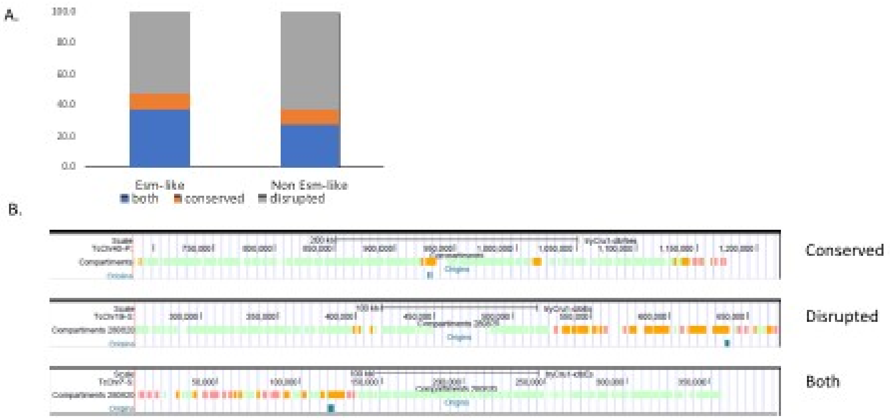

Additional file 2. Distribution of consensus replication origin regions located at DGF-1 genes according to their location at conserved (C- top) or disrupted (C-intermediate) *T. cruzi* genome domains (according to (43)). Both (B-below) represent DGF-1 contaning origins that could be in either one domain. B. Examples of replication origins domains at conserved, disrupted and in between both domains. According to (43), DGF-1, RHS and GP63 (orange) may be located either at disrupted (red) or conserved (blue)compartiments. The *T. cruzi* genome was loaded at UCSC genome browser and these 3 gene groups were colored in accordance. ORIs genome coordenates are shown in blue.
