# Supplementary material for: Replication origin location might contribute to genetic variability in Trypanosoma cruzi
Source: BMC Genomics. 2020 Jun 22;21:414. doi: 10.1186/s12864-020-06803-8 (PMC7310030; doi:10.1186/s12864-020-06803-8)
Supplement: Supplementary file 3 [file 12864_2020_6803_MOESM3_ESM.docx]

Additional file 3

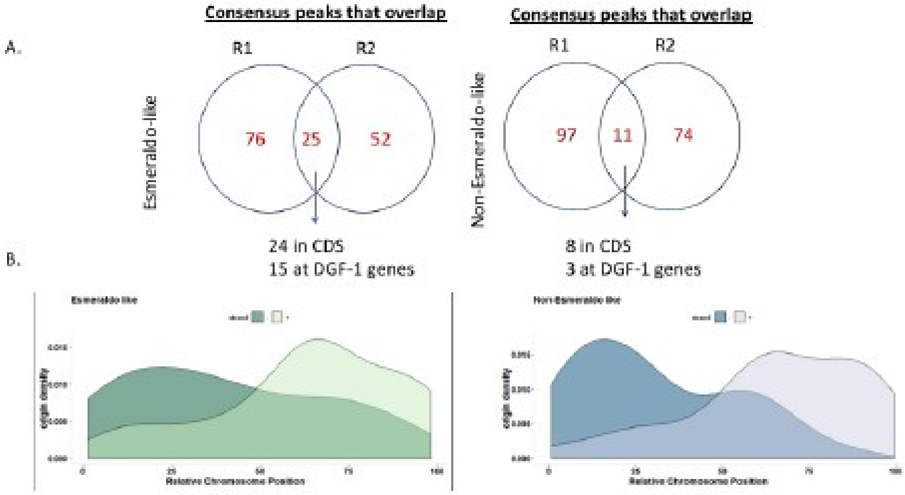

Additional file 3. Analysis of ORIs found at both R1 and R2 replicates. A. Consensus origin regions from replicates 1 (R1) and 2 (R2) were considered overlaped if they share at least one nucleotide. 60% (15/25) and 27% (3/11) of overlapped origins were located at DGF-1 genes in Esmeraldo-like and Non Esmeraldo like haplotype. B. Origins that were mapped at CDS were evaluated according their chromosome location.
