# Supplementary material for: Replication origin location might contribute to genetic variability in Trypanosoma cruzi
Source: BMC Genomics. 2020 Jun 22;21:414. doi: 10.1186/s12864-020-06803-8 (PMC7310030; doi:10.1186/s12864-020-06803-8)
Supplement: Supplementary file 4 [file 12864_2020_6803_MOESM4_ESM.docx]

Additional file 4

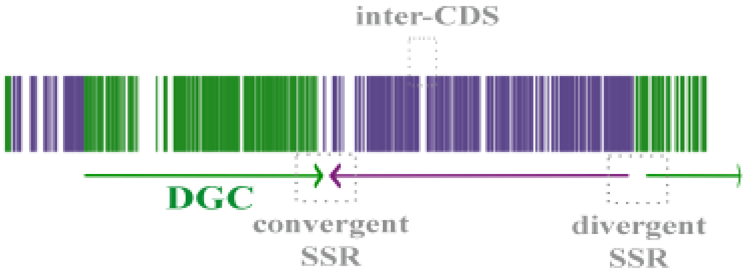

Additional file 4. Schematic representation of a *T. cruzi* genomic region. The CDSs are represented in green or purple vertical lines. The clusters of CDSs that are transcribed in the same direction represent a DGC. The arrows indicate the orientation of transcription. The green arrow is the transcription orientation of genes transcribed from the positive strand (green) or from the negative strand (purple).
