# Supplementary material for: Replication origin location might contribute to genetic variability in Trypanosoma cruzi
Source: BMC Genomics. 2020 Jun 22;21:414. doi: 10.1186/s12864-020-06803-8 (PMC7310030; doi:10.1186/s12864-020-06803-8)
Supplement: Supplementary file 5 [file 12864_2020_6803_MOESM5_ESM.docx]

Additional file 5

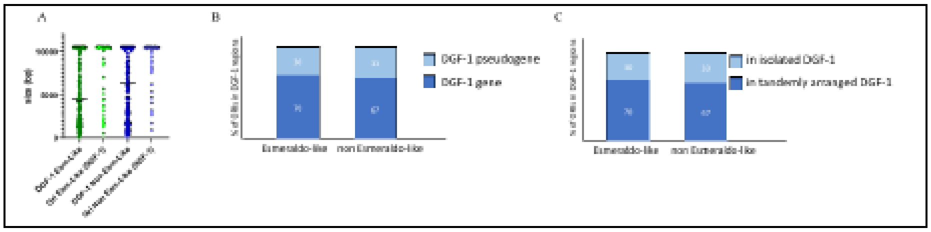

Additional file 5. Distribution of ORIs among DGF-1 genes. A. Scatter plot of DGF-1 genes distribution according their gene size (in bp). Total DGF-1 and DGF-1 containing replication origins were compared regarding their gene size. *P-value <0.005 – unpaired T-test Welch. B. Presence of ORIs in DGF-1 genes or pseudogenes. C. Presence of ORIs in DGF-1 genes/pseudogenes within DGFF-1 in tandemly arrays or in isolated DGF-1.
