# Supplementary material for: Replication origin location might contribute to genetic variability in Trypanosoma cruzi
Source: BMC Genomics. 2020 Jun 22;21:414. doi: 10.1186/s12864-020-06803-8 (PMC7310030; doi:10.1186/s12864-020-06803-8)
Supplement: Supplementary file 6 [file 12864_2020_6803_MOESM6_ESM.docx]

Additional file 6

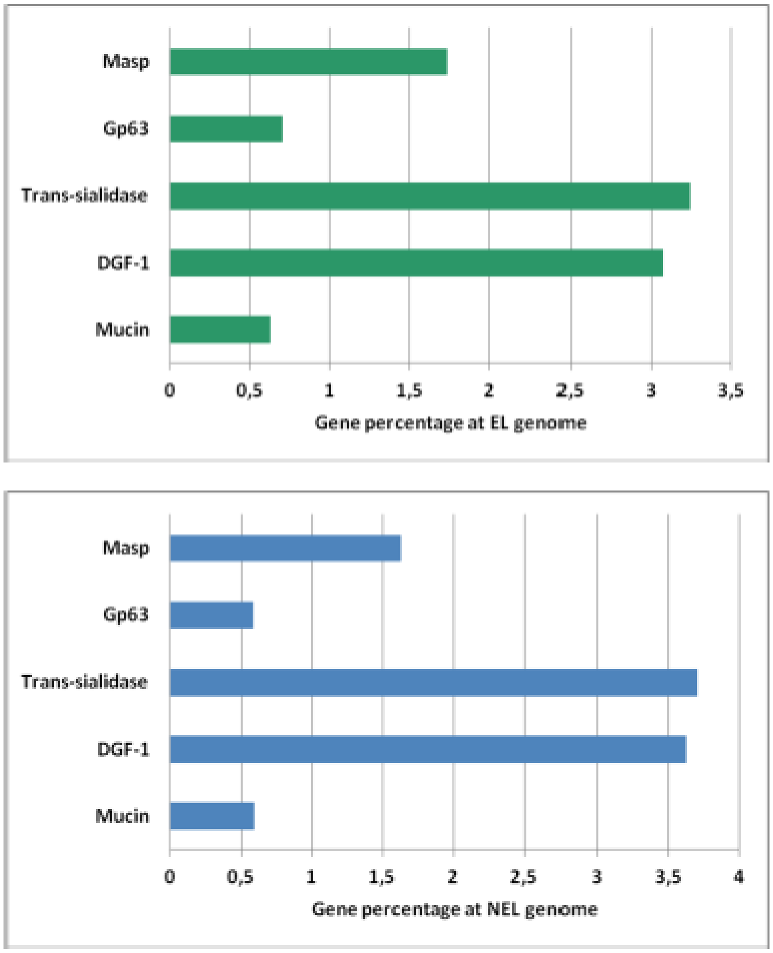

Additional file 6. Percentage of genes (in base pairs) in *T. cruzi* genome. The percentage of indicated genes belonging to the multigene family is represented for Sand P haplotypes. Sizes of genes in base pairs were normalized to the genome size.
