# Supplementary material for: Replication origin location might contribute to genetic variability in Trypanosoma cruzi
Source: BMC Genomics. 2020 Jun 22;21:414. doi: 10.1186/s12864-020-06803-8 (PMC7310030; doi:10.1186/s12864-020-06803-8)
Supplement: Supplementary file 7 [file 12864_2020_6803_MOESM7_ESM.docx]

Figure S7

A

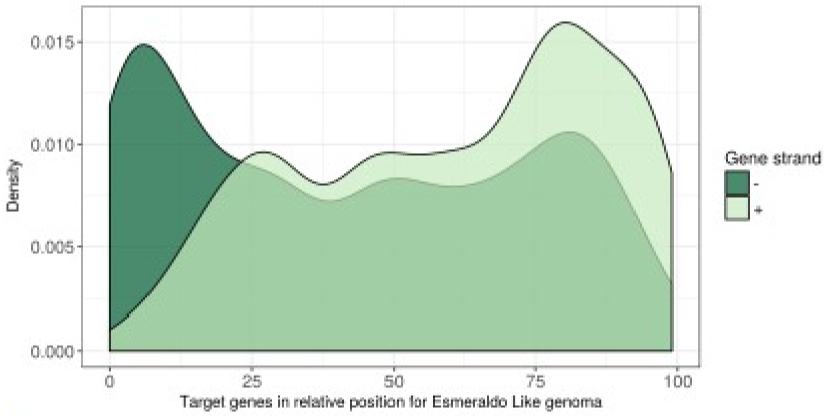

B

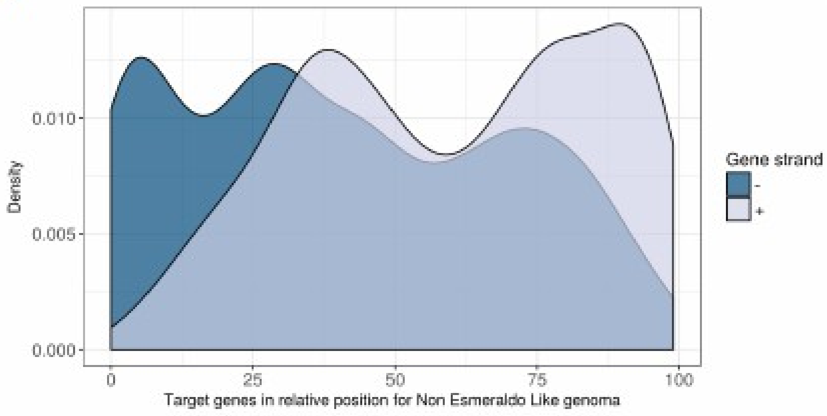

Additional file 7. Transcription orientation along chromosomes. Overall gene density and transcription orientation along each chromosome in S haplotype (A) and P haplotype (-) Genes transcribed in the negative strand; (+) genes transcribed in the positive strand.
